# Symptomatic small schwannoma is a risk factor for surgical complications and correlates with difficulty of enucleation

**DOI:** 10.1186/s40064-015-1547-9

**Published:** 2015-12-01

**Authors:** Kensaku Abe, Akihiko Takeuchi, Norio Yamamoto, Katsuhiro Hayashi, Kaoru Tada, Shinji Miwa, Hiroyuki Inatani, Yu Aoki, Takashi Higuchi, Hiroyuki Tsuchiya

**Affiliations:** Department of Orthopaedic Surgery, Graduate School of Medicine, Kanazawa University, 13-1 Takara-machi, Kanazawa, 920-8641 Japan

**Keywords:** Schwannoma, Clinical findings, Complication, Volume, Numbness, Location, Orthopaedic surgery

## Abstract

Postoperative neurological deficits of schwannomas are the complications that we want to avoid most. Predicting postoperative neurological deficits is crucial; however, the correlation between preoperative symptoms and neurological findings with postoperative neurological complications has not yet been completely clarified. Here we analyzed the risk factors for postoperative neurological complications. The study included 131 tumors from 107 patients histologically confirmed as schwannomas, which developed in the extremities and trunk without spinal cord involvement. The correlation between clinical findings and postoperative complications were statistically analyzed. One-hundred three tumors (78.6 %) had the preoperative neurological symptoms; these symptoms were detected in 93.3 % of small tumors (<4 cm^3^). We defined it as follows about the anatomical location of schwannomas. One is “central type” that normal nerve bundles widely splayed over the tumor’s capsule (tumor located in the central region of the nerve). Another is “peripheral type” that easy to enucleate without neurolysis (tumor located in the peripheral region of the nerve). Static analysis showed a significant difference in the Tinel sign, numbness, and postoperative neurological deficits (p = 0.04, 0.006, p < 0.001, respectively). Twenty-one cases (16.0 %) showed new postoperative neurological symptoms, including numbness in 12 cases, dysesthesia in three cases, pain in three cases, and slight motor palsy in two cases. In statistical analysis, small tumors (<4 cm^3^) significantly correlated with Tinel sign (p < 0.001), and was marginally significant with postoperative neurological deficits (p = 0.05). Moreover, small tumors (<4 cm^3^) accompanying numbness preoperatively significantly correlated with postoperative neurological deficits (p = 0.04). Small (<4 cm^3^) tumors significantly correlated with the preoperative neurological symptoms. Those tumors accompanying numbness also significantly correlated with the difficulty of the enucleation and postoperative neurological deficits. These findings will help to predict the neurological complication.

## Background

Schwannomas are benign neurogenic slow-growing tumors arising from Schwann cells (Gosk et al. 2004; Lee et al. 2001; Rosenberg et al. 1991). They were first described by Verocay in 1908 (Zhou et al. 2012). They are usually located around the craniocervical part at the surface or in an extremity. Schwannomas often develop at the age of 20–50 years and they are not associated with gender (Oberle et al. 1997; Pivlavaki et al. 2004; Rosenberg et al. 1991). Neurological symptoms such as the Tinel sign, numbness, and pain are typical clinical findings. Clinical symptoms that develop over time are mainly connected with compression of nerve fascicles (Jerzy et al. 2004; Rosenberg et al. 1991). Kwon et al. reported that there are few preoperative symptoms and signs in patients with intramuscular neurilemoma (Kwon et al. 2003). However, the etiologies have not yet been completely clarified. In contrast, postoperative neurological complications such as numbness, palsy, and sensory disturbances are severe problems. Kwon et al. also reported that patients with intramuscular schwannomas did not show the postoperative neurological functional deficits (Kwon et al. 2003). The incidence of postoperative palsy have been reported as 0.05–76.7 % (Kim et al. 2012; Lee et al. 2001; Sawada et al. 2006). Although predicting postoperative neurological deficits is very important, the risk factors for postoperative neurological deterioration have not yet been discussed. The purpose of this study was to investigate the clinical features of symptomatic schwannoma and to analyze the risk factors for postoperative neurological complications.

## Methods

### Patients and methods

**Fig. 1:**
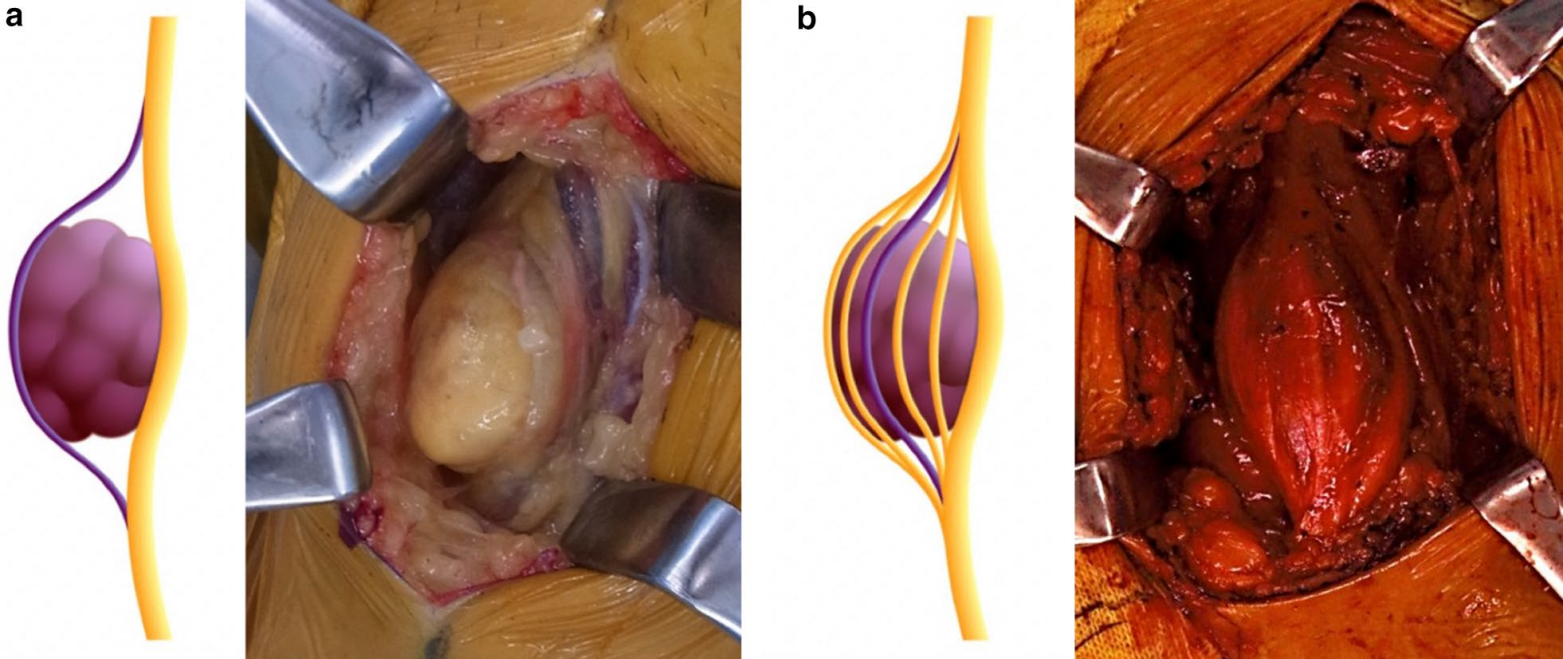
Location of the tumor from the intraoperative findings. **a** Peripheral type [easy to enucleate without neurolysis (tumor located in the peripheral region of the nerve)]. **b** Central type [normal nerve bundles widely splayed over the tumor capsule (tumor located in the central region of the affected nerve)]

This study was a retrospective cohort study included 131 schwannomas from 107 patients, were operated from 2002 to 2014. The mean age of the patients was 49 years (range 15–80 years), and the mean follow-up period was 10.1 months (range 1–76 months). Ninety-four patients had solitary schwannoma and 11 had schwannomatosis. Patients were identified by retrospectively searching the hospital’s database for histologically diagnosed schwannoma. Tumors involving the extremity and trunk were included, however tumors in spinal cord were excluded. The preoperative neurological symptoms and postoperative neurological deficits were evaluated from the medical records. The location and the volume of the tumor were analyzed using the preoperative magnetic resonance imaging (MRI). All of the surgery was performed in Kanazawa university hospital. After the exposure of tumor capsule, the normal nerve bundles over the tumor capsule were detected using the nerve stimulator. And then, a longitudinal incision was carefully made in the epineurium with avoiding the normal nerve bundle and the onion skin like thin epineurium was peeled off gently from the tumor. Finally, these procedures allowed to enucleate the tumor. The schwannomas were usually easily enucleated without the complicated neurolysis. However, in some cases the complicated neurolysis were needed when the normal nerve bundles widely splayed over the tumor’s capsule. So, we newly defined the tumor location from intraoperative findings: peripheral type [easy to enucleate without neurolysis (tumor located in the peripheral region of the nerve)] and central type [normal nerve bundles widely splayed over the tumor’s capsule (tumor located in the central region of the nerve)] (Fig. 1). Tumor volume was calculated using the following formula (Tomayko and Reynolds 1989): π/6 × L × W × H (L, length; W, width; H; height).

### Statisitical analysis

Statistical analysis was performed with Chi-square test and logistic analysis using SPSS ver. 19.0 (SPSS Inc., Chicago, IL, USA); p < 0.05 was considered to indicate statistical significance.

## Results

One-hundred three of the 131 tumors (78.6 %) produced preoperative neurological symptoms. Tinel sign was observed in 77 (58.8 %), spontaneous pain in 32 (24.4 %), tenderness in 58 (44.3 %), and numbness in 38 tumors (29.0 %). Chi-square test revealed a correlation of spontaneous pain with the extremity location (p = 0.045) and the small tumor (<4 cm^3^, p = 0.029). No significant differences were observed with regard to tenderness. Chi-square test and logistic analysis showed a significant difference in Tinel sign between the tumor volumes (<4 and ≥4 cm^3^; p < 0.001 for both), major nerve and peripheral nerve (p < 0.001 and p = 0.001, respectively), and central type and peripheral type (p = 0.004 and 0.047, respectively). Further, a significant difference in numbness between major nerve and peripheral nerve (p < 0.001 and p = 0.024, respectively), and the central type and peripheral type (p < 0.001 and p = 0.006, respectively) was observed. Tumors located intermuscularly and originating from a major nerve also correlated with numbness (Table 1a–d). Taken together, these results suggested that small tumor significantly correlated with the neurological symptoms (spontaneous pain and Tinel sign).

**Table 1:** Correlation of neurological symptoms with each factors

	Spontaneous pain		X^2^ test	Logistic analysis		
	Y	N	P value	OR	95 % CI	P value
(a)						
Gender						
Male	16	58	0.394	0.725	0.307–1.713	0.463
Female	16	41		1		
Age (years)						
≥50	15	52	0.578	0.706	0.297–1.678	0.431
<50	17	47		1		
Location						
Extremity	31	82	0.045	4.388	0.530–36.331	0.17
Trunk	1	17		1		
Nerve origin						
Major	20	52	0.324	1.548	0.624–3.840	0.346
Minor	12	47		1		
Muscular location						
Intermuscle	23	81	0.227	0.47	0.170–1.296	0.144
Intramuscle	9	18		1		
Neuro-location						
Peripheral	27	79	0.567	1		
Central	5	20		0.794	0.251–2.509	0.694
Volume (cm^3^)						
<4	20	40	0.029	2.141	0.893–5.131	0.088
≥4	12	59		1		
Total	32	99				

	Tenderness		X^2^ test	Logistic analysis		
	Y	N	P value	OR	95 % CI	P value
(b)						
Gender						
Male	30	44	0.327	0.693	0.332–1.447	0.329
Female	28	29		1		
Age (years)						
≥50	26	41	0.197	0.536	0.255–1.126	0.1
<50	32	32		1		
Location						
Extremity	53	60	0.129	2.221	0.692–7.134	0.18
Trunk	5	13		1		
Nerve origin						
Major	34	38	0.453	1.288	0.596–2.782	0.519
Minor	24	35		1		
Muscular location						
Intermuscle	46	58	0.984	0.835	0.335–2.082	0.698
Intramuscle	12	15		1		
Neuro-location						
Peripheral	46	60	0.677	1		
Central	12	13		1.216	0.472–3.131	0.686
Volume (cm^3^)						
<4	28	32	0.612	1.023	0.487–2.152	0.951
≥4	30	41		1		
Total	58	73				

	Tinel sign		X^2^ test	Logistic analysis		
	Y	N	P value	OR	95 % CI	P value
(c)						
Gender						
Male	42	32	0.592	0.674	0.264–1.721	0.409
Female	35	22		1		
Age (years)						
≥50	42	25	0.353	1.424	0.574–3.534	0.446
<50	35	29		1		
Location						
Extremity	73	40	0.001	3.683	0.933–14.538	0.063
Trunk	4	14		1		
Nerve origin						
Major	55	17	<0.001	4.67	1.846–11.816	0.001
Minor	22	37		1		
Muscular location						
Intermuscle	66	38	0.033	1.914	0.647–5.663	0.241
Intramuscle	11	16		1		
Neuro-location						
Peripheral	56	50	0.004	1		
Central	21	4		3.772	1.018–13.984	0.047
Volume (cm^3^)						
<4	47	13	<0.001	6.594	2.487–17.480	<0.001
≥4	30	41		1		
Total	77	54				

	Numbness		X^2^ test	Logistic analysis		
	Y	N	P value	OR	95 % CI	P value
(d)						
Gender						
Male	19	55	0.338	0.601	0.244–1.479	0.268
Female	19	38		1		
Age (years)						
≥50	21	46	0.547	1.072	0.442–2.600	0.878
<50	17	47		1		
Location						
Extremity	35	78	0.214	1.52	0.344–6.720	0.581
Trunk	3	15		1		
Nerve origin						
Major	30	42	<0.001	3.04	1.157–7.989	0.024
Minor	8	51		1		
Muscular location						
Intermuscle	36	68	0.006	4.581	0.954–21.996	0.057
Intramuscle	2	25		1		
Neuro-location						
Peripheral	23	83	<0.001	1		
Central	15	10		4.209	1.517–11.681	0.006
Volume (cm^3^)						
<4	20	40	0.316	1.611	0.654–3.971	0.3
≥4	18	53		1		
Total	38	93				

	Postoperative deficits		X^2^ test	Logistic analysis		
	Y	N	P value	OR	95 % CI	P value
(e)						
Gender						
Male	13	61	0.585	0.824	0.226–3.001	0.769
Female	8	49		1		
Age (years)						
≥50	9	58	0.407	0.479	0.137–1.670	0.248
<50	12	52		1		
Location						
Extremity	17	96	0.441	0.171	0.026–1.132	0.067
Trunk	4	14		1		
Nerve origin						
Major	16	56	0.033	3.92	0.860–17.861	0.078
Minor	5	54		1		
Muscular location						
Intermuscle	17	87	0.847	0.228	0.046–1.138	0.071
Intramuscle	4	23		1		
Neuro-location						
Peripheral	7	99	<0.001	1		
Central	14	11		32.04	7.566–135.704	<0.001
Volume (cm^3^)						
<4	12	48	0.255	3.742	0.989–14.160	0.052
≥4	9	62		1		
Total	21	110				

*Y* yes, *N* no, *OR* odds ratio, *CI* confidential interval

The postoperative neurological deficits were detected in 21 cases (16 %), including numbness in 14 cases, dysesthesia in three cases, pain in three cases, and slight motor palsy in one case. One case complained of numbness and pain simultaneously. Numbness that newly developed postoperatively was ameliorated in three cases, and postoperative dysesthesia and pain disappeared in all the cases after the mean term of 7.2 months (range 1–69 months). Although incomplete motor nerve palsy persisted in one case, he showed no restrictions in his daily life. Chi-square test and logistic analysis further showed that the central type significantly correlated with postoperative neurological deficits (p < 0.001). Chi-square test showed that tumors originating from a major nerve significantly correlated with a postoperative neurological deficit (p = 0.033) and logistic analysis showed that those showed marginal significance to produce it (p = 0.078) (Table 1e). Moreover, small tumor (<4 cm^3^) accompanying numbness significantly correlated with postoperative neurological deficits (p = 0.04 in Chi-square test and p = 0.038 in logistic analysis) (Table 2a, b).

**Table 2:** Relationship between preoperative neurological symptoms and new postoperative neurological deficits

	All tumors (n = 131)					
	Postoperative deficits		X^2^ test	Logistic analysis		
	Y	N	P value	OR	95 % CI	P value
(a)						
Spontaneous pain						
Y	5	27	0.943	0.923	0.293–2.910	0.891
N	16	83		1		
Tenderness						
Y	10	48	0.736	1.149	0.426–3.095	0.784
N	11	62		1		
Tinel sign						
Y	14	63	0.423	1.142	0.387–3.370	0.809
N	7	47		1		
Numbness						
Y	9	29	0.127	1.975	0.693–5.629	0.203
N	12	81		1		

	Small tumors (<4 cm^3^, n = 60)					
	Postoperative deficits		X^2^ test	Logistic analysis		
	Y	N	P value	OR	95 % CI	P value
(b)						
Spontaneous pain						
Y	4	16	1	0.944	0.217–4.106	0.938
N	8	32		1		
Tenderness						
Y	6	22	0.796	1.216	0.308–4.798	0.78
N	6	26		1		
Tinel sign						
Y	9	38	0.754	0.516	0.100–2.659	0.429
N	3	10		1		
Numbness						
Y	7	13	0.04	4.378	1.088–17.609	0.038
N	5	35		1		

*Y* yes, *N* no, *OR* odds ratio, *CI* confidential interval

The difficulty of the enucleation was due to the degree of the normal nerve bundle over the tumor capsule, which needs to be detached. In this study, we categorized the tumor location into two groups: peripheral and central type based on these operative findings. Chi-square test showed that central location significantly correlated with Tinel sign (p = 0.004) and numbness (p < 0.001). Logistic analysis detected the significant correlation between the central location and numbness (p = 0.007, Table 3). These results suggested that schwannoma accompanying numbness correlated with the difficulty of the enucleation. As mentioned in the second question, numbness in small tumor (<4 cm^3^) also correlated with postoperative neurological deficits.

**Table 3:** Relationship between preoperative neurological symptoms and tumor location

	All tumors (n = 131)					
	Tumor location		X^2^ test	Logistic analysis		
	Peropheral	Central	P value	OR	95 % CI	P value
Spontaneous pain						
Y	27	5	0.567	0.696	0.217–2236	0.543
N	79	20		1		
Tenderness						
Y	46	12	0.677	1.097	0.414–2.901	0.853
N	60	13		1		
Tinel sign						
Y	56	21	0.004	2.893	0.854–9.801	0.088
N	50	4		1		
Numbness						
Y	23	15	<0.001	3.847	1.440–10,274	0.007
N	83	10		1		

*Y* yes, *N* no, *OR* odds ratio, *CI* confidential interval

## Discussion

Schwannomas are benign neurogenic slow-growing tumors with typical neurological symptoms such as Tinel sign, numbness, and pain (Zhou et al. 2012). In contrast, postoperative neurological complications such as numbness, palsy, and sensory disturbances are severe problems, which have been reported as 0.05–76.7 % (Kim et al. 2012; Lee et al. 2001; Sawada et al. 2006). Although surgical complication might be due to the difficulty of enucleation, the correlation of operative findings with neurological symptoms and surgical complications were not fully discussed. In this study, we detected the small (<4 cm^3^) tumor tended to accompany the neurological symptoms and the preoperative numbness correlated with the difficulty of enucleation. Moreover, small tumor accompanying numbness had the strong impact on and the postoperative neurological deficit. These new findings will be helpful to predict the surgical complications.

We found that tumors developing in the extremities or those with a small (<4 cm^3^) volume correlated with spontaneous pain and Tinel sign. Tumors originating from a major nerve or those that were located in the central region of the affected nerve correlated with Tinel sign and numbness. Regarding to the timing of the onset of the symptoms, we considered that the location of tumor might influence on it. We speculate that if a tumor affects the surrounding unaffected nerves due to its location (central type) or any other reason, then the patient notices the tumor mass early, resulting in diagnosis when the tumor volume is still small. However, in a case of slow glowing without influencing any surrounding nerves, then patient is unable to notice the tumor until its size considerably increases.

Postoperative neurological complications such as numbness, palsy, and sensory disturbances are severe problems, which have been reported as 0.05–76.7 % (Kim et al. 2012; Lee et al. 2001; Sawada et al. 2006). Park et al. reported that 73.2 % of all tumors produce postoperative neurological symptoms and that the symptoms persist in 30 % cases (Park et al. 2009). They also mentioned that a preoperative needle biopsy should not be performed for diagnosis to prevent the injury of nerve. Godwin reported that a main nerve involving the brachial plexus cord was sacrificed in three of 14 patients, thus resulting in permanent damage (Godwin 1952). He also reported patients who underwent unnecessary radical resection because the needle biopsy was misinterpreted as a pleomorphic pattern or malignancy. Lee et al. reported that a pleomorphic pattern was observed in a patient after an incisional biopsy, which suggested malignancy, and a radical resection was performed; thus, an incisional biopsy may be recommended in unusual cases (Lee et al. 2001). They also reported that the postoperative complications were observed in 15 of 78 patients. Postoperative paresthesia, which was the most common complication, occurred in seven patients and muscle weakness in three; five of ten patients recovered completely within a year, which they believed was because iatrogenic nerve damage was incomplete. Whitaker and Droulias reported that temporary muscle weakness or diminished sensory perception occurs after dissecting nerve fibers (Whitaker and Droulias 1976). Kehoe reported postoperative issues, including severe hemorrhage after exploration of the brachial plexus and pain at the operative site. A motor-nerve deficit, sensory-nerve deficit, and mixed-nerve deficit were noted in 13 of 79 adequately recorded cases, indicative of that careful clinical and imaging examinations are essential (Kehoe et al. 1995). The possibility of iatrogenic nerve injury at the time of exploration should be considered and discussed with the patient preoperatively. The nerve must be clearly exposed both proximally and distally to the site of the lesion.

In this study, the postoperative neurological deficits were detected in 21 cases (16 %) and the sensory disturbance such as numbness, postoperative dysesthesia and pain ameliorated in all the cases except one case of incomplete motor nerve palsy. The tumors originating from a major nerve significantly correlated with a postoperative neurological deficit in univariate analysis (p = 0.033), however multivariate analysis lost its significance (p = 0.078). Small tumor (<4 cm^3^) accompanying numbness had the strong impact on postoperative neurological deficits not only in univariate analysis (p = 0.04) but also in multivariate analysis (p = 0.038). This study also showed a significant difference between tumors located in the central region of the nerve and postoperative neurological deficits. However, this was a subjective evaluation, which was determined during surgery by different physicians. Thus, the impact of central type of tumors on postoperative neurological deficits is weaker than other objective factors such as tumor size or the nerves involved. We expect that advances in imaging technology should help evaluate tumor location in the nerve more clearly.

Kim et al. reported that larger tumors tend to produce a greater risk of postoperative neurological deficits, because these tumors seem to have a higher frequency of fascicular injury during dissection (Kim et al. 2012). We considered that the difficulty of the enucleation was due to the degree of the normal nerve bundle over the tumor capsule, which needs to be detached. In this study, we categorized the tumor location into two groups: peripheral and central type based on these operative findings. Chi-square test showed that central location significantly correlated with Tinel sign (p = 0.004) and numbness (p < 0.001). Logistic analysis detected the significant correlation between the central location and numbness (p = 0.007). These results suggested that schwannoma accompanying numbness correlated with the difficulty of the enucleation. As mentioned in the second question, numbness in small tumor (<4 cm^3^) also correlated with postoperative neurological deficits. In such tumors, the use of microscope might contribute to facilitate the normal nerve and to prevent the postoperative neurological deficit (Omezzine et al. 2009).

This study had a number of limitations. First, this study was a retrospective cohort study involving surgically excised schwannomas, and some factors related to different physicians may have affected the results. Second, the incidence of schwannoma is relatively low; thus, we included relatively few cases, and it was difficult to draw clear conclusions. However, this study was conducted only in one institute, and it is different, but the doctor had the similar strategy, and the passable number of 100 cases gathered while there was few it. So we believe that the limitations do not jeopardize our conclusion.

## Conclusions

Symptomatic small schwannoma especially accompanying numbness is a novel risk factor for postoperative neurological deficits and also correlates with difficulty of enucleation. Although further analysis is necessary, these findings will be helpful to predict the neurological complications based on preoperative clinical findings.
